# LiteMamba-Synth: lightweight state space models for efficient 3T-to-7T MRI translation

**DOI:** 10.3389/fnana.2026.1886476

**Published:** 2026-07-09

**Authors:** Zhengrui Zhang, Jie Dong, Haoting Yang, Zehong Chen

**Affiliations:** 1 School of Computer Science and Engineering, Huizhou University, Huizhou, China; 2 School of Mathematics and Statistics, Huizhou University, Huizhou, China

**Keywords:** deep learning, lightweight, medical imaging, MRI translation, parameter efficiency, state space models

## Abstract

**Introduction:**

The superior clinical utility of 7T magnetic resonance imaging (MRI) is constrained by high acquisition costs and limited scanner availability. While deep learning-based 3T-to-7T synthesis offers a potential solution, prevailing models typically rely on heavy parameterization, which increases computational redundancy and risk of overfitting on restricted medical datasets. In this paper, we focus on model efficiency and propose LiteMamba-Synth, an architecturally streamlined state space framework designed for high-fidelity MRI translation with minimal resource requirements.

**Methods:**

Our core contribution is the integration of the ConvMamba block, which utilizes the linear-time complexity of State Space Models (SSMs) to capture expansive spatial dependencies without the prohibitive computational overhead of traditional attention mechanisms. To preserve essential anatomical details during the compression of the feature space, we introduce the Wavelet-Enhanced Skip connection (WES), a module that facilitates multi-scale frequency-domain feature fusion to safeguard high-frequency textures and edge information. Additionally, a lightweight Convolutional Block Attention Module (CBAM) is incorporated to adaptively recalibrate feature responses toward salient neuroanatomical regions.

**Results:**

Experimental results on the UNC T1w dataset demonstrate that LiteMamba-Synth achieves a competitive PSNR of 20.82 dB and an SSIM of 0.711. Crucially, our model maintains a compact footprint of merely 2.15 million parameters, representing a substantial reduction in complexity compared to contemporary deep learning baselines.

**Discussion:**

By delivering high-quality synthesis results with minimal parameter overhead, LiteMamba-Synth provides a practical and scalable solution for deploying advanced MRI synthesis in resource-constrained clinical environments.

## 1 Introduction

Ultra-high-field (UHF) 7T magnetic resonance imaging (MRI) has demonstrated transformative clinical utility in neurodiagnostic and pathological assessments—such as Alzheimer's disease and cardiovascular disorders—owing to its exceptional signal-to-noise ratio (SNR) and superior spatial resolution (van der Kolk et al., 2013). Nevertheless, the widespread integration of 7T systems into routine clinical practice is severely circumscribed by exorbitant acquisition costs and prohibitive maintenance requirements. In recent years, the advancement of deep learning has catalyzed the emergence of 3T-to-7T MRI synthesis as a cost-effective computational alternative (Qu et al., 2020). By leveraging neural networks to reconstruct simulated 7T images from standard 3T scans, this paradigm facilitates the recovery of intricate pathological details and texture fidelity that are otherwise indiscernible in 3T acquisitions, offering a viable and innovative trajectory for high-quality clinical neuroimaging.

### 1.1 Current situation and challenges

The domain of medical image synthesis has witnessed the exploration of diverse deep learning paradigms. Early research predominantly revolved around Generative Adversarial Networks (GANs), with frameworks such as CycleGAN (Zhu et al., 2017), pix2pix (Isola et al., 2017), and RegGAN (Kong et al., 2021) being proposed to achieve high-fidelity pathological image generation. Subsequently, Convolutional Neural Network (CNN)-based methods introduced enhancement mechanisms like wavelet transforms (Mallat, 1989), leading to the development of lightweight architectures such as DDCNN (Zhang et al., 2018) and WATNet (Qu et al., 2020), which further refined local feature extraction capabilities. More recently, Transformer architectures have been extensively integrated due to their robust long-range dependency modeling—exemplified by ResViT (Dalmaz et al., 2022) and PTNet (Zhang et al., 2022)—significantly elevating the quality of 3T-to-7T translation. This trend is driven by the success of Vision Transformers (Dosovitskiy et al., 2021) and Swin Transformers (Liu et al., 2021) in capturing long-range spatial dependencies, which are essential for medical image attention modeling. This success is largely attributed to the spatial recalibration capabilities inherited from classic attention mechanisms (Jaderberg et al., 2015), which allow models to focus on salient anatomical regions. Additionally, State Space Models (SSMs) (Gu and Dao, 2023) have gained increasing traction for their linear-time complexity; approaches like Restore-RWKV (Yang et al., 2025) and FS-RWKV (Lei et al., 2025) have introduced new possibilities for computationally efficient synthesis.

Despite these advancements, a critical trade-off persists in contemporary methodologies. Existing frameworks either suffer from prohibitive parameterization, which triggers overfitting on sparse medical datasets, or adopt excessive lightweight strategies that compromise anatomical texture fidelity. This challenge has prompted the exploration of diverse attention variants to balance efficiency and representation power (Hou et al., 2021). To bridge this gap, we present LiteMamba-Synth, an architecturally streamlined state space framework that strikes an optimal balance between parameter efficiency and synthesis quality.

### 1.2 Summary of contributions

The main contributions of this article can be summarized as follows:

- **First, we introduce LiteMamba-Synth**, which, to the best of our knowledge, represents the initial integration of an architecturally streamlined State Space Model (SSM) (Zhu et al., 2024) with linear-time complexity into the domain of 3T-to-7T MRI translation. Diverging from contemporary deep learning paradigms that rely on excessively large parameter scales, LiteMamba-Synth is engineered with computational efficiency as its foundational principle. This work establishes a novel lightweight synthesis framework specifically optimized for small-sample medical imaging cohorts, effectively mitigating the risk of overfitting inherent in large-scale models.
- **Second, to preserve high reconstruction fidelity within a compact footprint**, we propose a synergistic design comprising three core modules:

- **ConvMamba block:** Leveraging the scaling advantages of SSMs (Gu and Dao, 2023), this block captures expansive global spatial dependencies while circumventing the quadratic computational overhead characteristic of traditional attention mechanisms.
- **Wavelet-enhanced skip connection (WES):** Transcending the limitations of standard U-Net skip connections (Ronneberger et al., 2015), WES orchestrates multi-scale frequency-domain feature fusion (Liu et al., 2018) across the L1–L3 encoding-decoding hierarchies, significantly reinforcing the transmission and retention of high-frequency anatomical textures and edge-wise integrity.
- **Integrated CBAM:** By employing a dual-pathway attention mechanism (Hu et al., 2018; Oktay et al., 2018), the Convolutional Block Attention Module (CBAM) (Woo et al., 2018) adaptively recalibrates feature responses, prioritizing salient neuroanatomical regions to further elevate synthesis precision.

Driven by these coordinated innovations, LiteMamba-Synth achieves highly competitive performance using an exceptionally lean footprint of merely 2.15M parameters, demonstrating a superior trade-off between synthesis quality and hardware efficiency.

- **Third, rigorous empirical evaluation on the UNC T1w dataset** substantiates the efficacy of our proposed framework. LiteMamba-Synth achieves a Peak Signal-to-Noise Ratio (PSNR) of 20.82 dB and a Structural Similarity Index (SSIM) of 0.711. Remarkably, while constrained to a compact footprint of merely 2.15M parameters, our model demonstrates superior synthesis fidelity compared to established representative baselines, including CycleGAN, pix2pix, ResViT, and FS-RWKV. These results empirically validate that LiteMamba-Synth successfully circumvents the common performance degradation associated with model compression, delivering high-fidelity MRI translation through an optimal balance of computational parsimony and reconstruction precision.

## 2 Materials and methods

### 2.1 Dataset and pre-processing

This study conducted performance evaluation using the publicly available BNU-UNC paired dataset, which contains 3T and 7T brain T1-weighted MRI images from 30 subjects. The UNC site data served as the primary validation platform. All original images underwent a standardized preprocessing pipeline. First, the N4 bias field correction algorithm (Tustison and Gee, 2009) was applied to mitigate the effects of magnetic field inhomogeneity. The imaging model can be expressed as:


$$\begin{array}{l} v\left(x\right)=u\left(x\right)\cdot b\left(x\right)+n\left(x\right) \end{array}$$ (1)

where *v*(*x*) denotes the observed image, *u*(*x*) is the underlying true image to be restored, *b*(*x*) represents the bias field, and *n*(*x*) is additive noise. Subsequently, the 3T and 7T images were aligned via rigid (Avants et al., 2011) registration to ensure spatial correspondence of anatomical structures. To eliminate intensity discrepancies across different scanning protocols, min–max normalization was applied, scaling all pixel intensities to the interval [0, 1] according to:


$$\begin{array}{l} x_{\text{norm}}=\frac{x-x_{\min}}{x_{\max}-x_{\min}} \end{array}$$ (2)

where *x*_max_ and *x*_min_ denote the maximum and minimum pixel intensities of the original image, respectively. After preprocessing, the volumetric data were sliced axially, yielding a training set of approximately 1,600 two–dimensional slices. To prevent data leakage and ensure unbiased evaluation, the validation and test sets were partitioned under a strict subject–level independence protocol.

### 2.2 LiteMamba-Synth architecture

**Figure 1:**
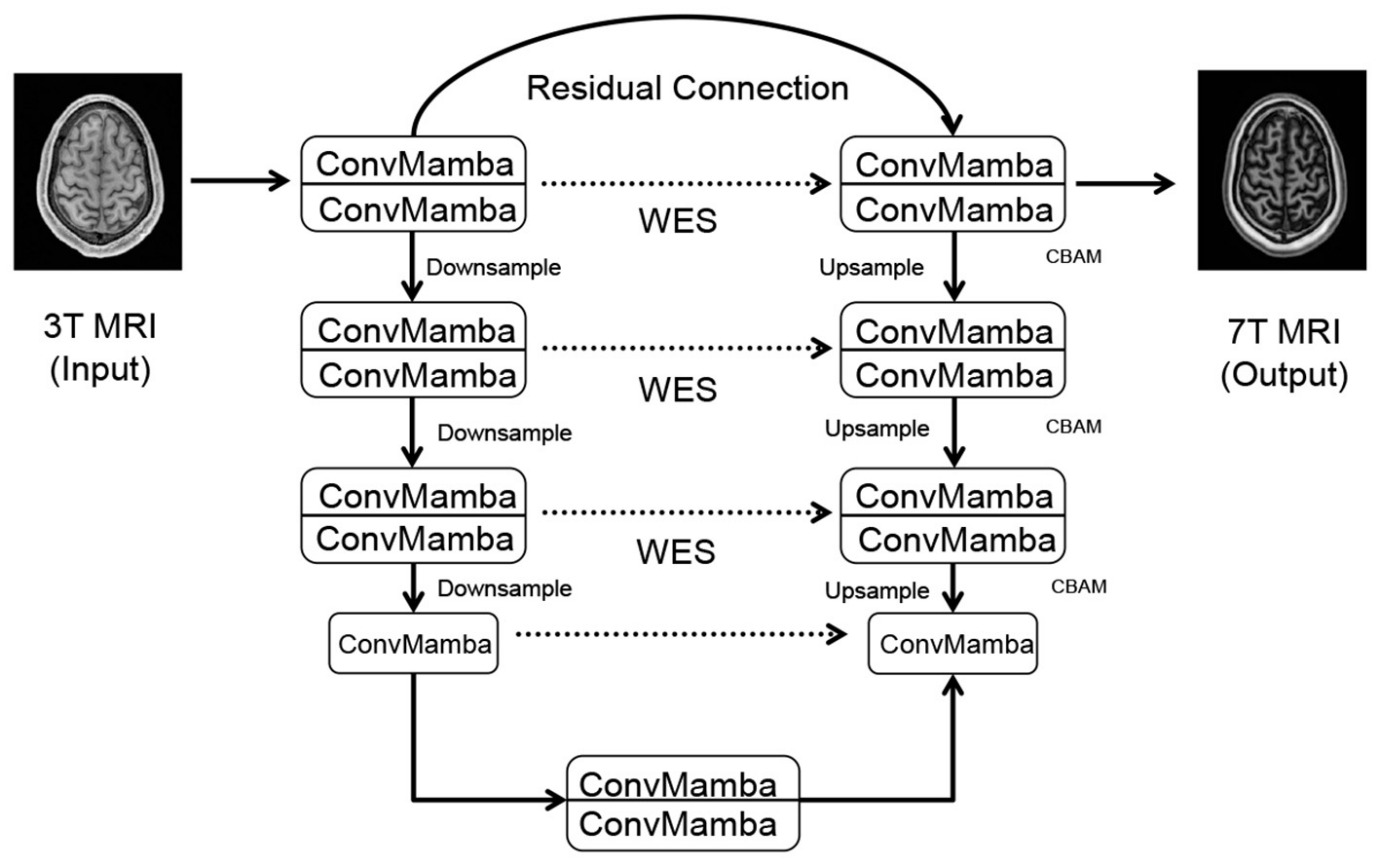
Overall architecture of LiteMamba-Synth.

The proposed LiteMamba-Synth framework is built upon a hierarchical U-Net-like backbone, as illustrated in Figure 1. Using a single-channel 3T MRI slice as the input, the network performs multi-scale feature extraction and hierarchical decoding through a four-level cascade of ConvMamba modules (L1 to L4). This process facilitates the high-fidelity synthesis of 7T MR images, maintaining the original spatial resolution and dimensions of the input. The architectural design centers on three principal components: the ConvMamba block, the Wavelet-Enhanced Skip connection (WES), and the Convolutional Block Attention Module (CBAM).

#### 2.2.1 ConvMamba block

As the core computational backbone, the ConvMamba block is designed to bypass the quadratic complexity inherent in standard Transformer-based self-attention. By leveraging the linear-time efficiency of State Space Models (SSMs), this module effectively characterizes long-range spatial correlations while significantly reducing computational overhead. For an input feature map **X**∈ℝ^*B*×*C*×*H*×*W*^, the block employs two successive residual operations. The first residual branch integrates LayerNorm (LN) (Ba et al., 2016), depthwise convolution (DWConv), SiLU activation (Elfwing et al., 2018), and a 1 × 1 convolution:


$$\begin{array}{l} {\text{X}}_{1}=\text{Con}{\text{v}}_{1\times 1}(\text{SiLU}(\text{DWConv}\left(\text{LN}\left(\text{X}\right)\right)))+\text{X}. \end{array}$$ (3)

The second residual branch refines the representation using LayerNorm followed by a two-layer MLP with an expansion ratio of 4 and GELU activation (Hendrycks and Gimpel, 2016):


$$\begin{array}{l} {\text{X}}_{2}=\text{MLP}(\text{LN}\left({\text{X}}_{1}\right))+{\text{X}}_{1}, \end{array}$$ (4)

where $\text{MLP}\left(\cdot \right)=\text{Con}{\text{v}}_{1\times 1}^{C\to 4C}\left(\cdot \right)\to \text{GELU}\to \text{Con}{\text{v}}_{1\times 1}^{4C\to C}\left(\cdot \right)$. By eschewing the quadratic complexity of traditional attention, the ConvMamba block achieves efficient global context aggregation, making it particularly suitable for processing high-resolution medical imaging data.

#### 2.2.2 Wavelet-enhanced skip connection

To mitigate the attenuation of high-frequency components during the encoding process, we incorporate a Wavelet-Enhanced Skip connection (WES) across the three hierarchical skip levels (L1 to L3). The WES module employs a Haar wavelet decomposition to partition the encoder feature map **E**∈ℝ^*C*×*H*×*W*^ into four distinct sub-bands: **E**_*LL*_, **E**_*LH*_, **E**_*HL*_, and **E**_*HH*_. To selectively amplify textural details, the high-frequency sub-bands are modulated by learnable channel-wise coefficients $\gamma_{sub}\in {\mathbb{R}}^{C}$, constrained within [−1, 1] via the tanh function:


$$\begin{array}{l} {\tilde{\text{E}}}_{sub}=\tanh\left(\gamma_{sub}\right)⊙{\text{E}}_{sub},\;sub\in \left\{LH,HL,HH\right\}. \end{array}$$ (5)

Following the inverse wavelet transform (IWT) for reconstruction, the enhanced feature map $\tilde{\text{E}}$ is further refined by integrating a weighted residual term:


$$\begin{array}{l} {\text{E}}_{\text{WES}}=\tilde{\text{E}}+\alpha \cdot \left(\tilde{\text{E}}-{\text{E}}_{LL}^{↑}\right), \end{array}$$ (6)

where ${\text{E}}_{LL}^{↑}$ represents the bilinearly upsampled low-frequency approximation and α = 0.1 serves as a stability constant. This architecture facilitates the restoration of sharp edges and intricate anatomical textures that are typically compromised during pooling operations.

#### 2.2.3 Convolutional block attention module

**Figure 2:**
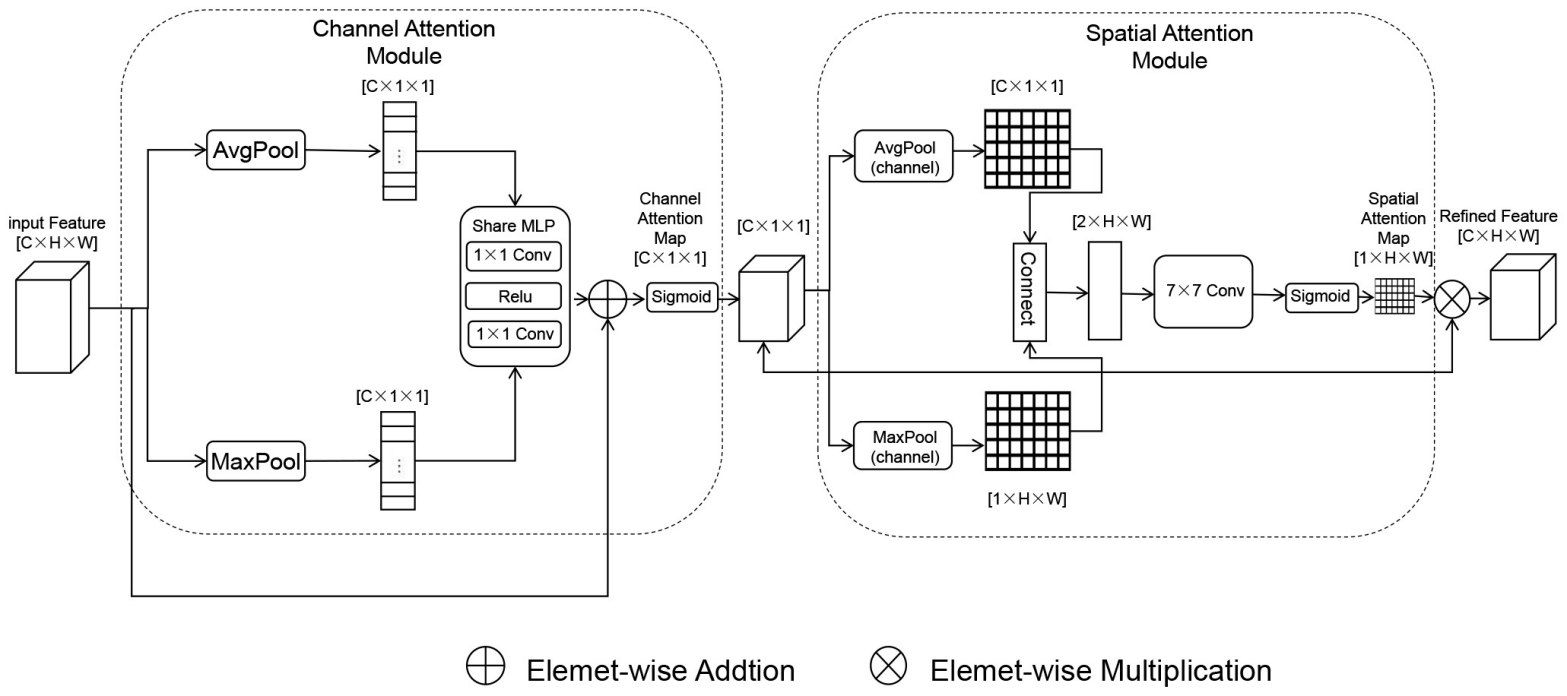
Structure of the CBAM module. Channel attention (top branch) and spatial attention (bottom branch) are cascaded to refine the feature map F ∈ ℝ^*C*×*H*×*W*^.

To further refine the feature representations, a lightweight Convolutional Block Attention Module (CBAM) is embedded at the terminus of each ConvMamba block within the decoder stages. The internal structure of CBAM is illustrated in Figure 2. This module sequentially executes channel and spatial attention (Hu et al., 2018; Oktay et al., 2018) to recalibrate the intermediate feature map **F**∈ℝ^*C*×*H*×*W*^. Specifically, the channel attention component aggregates global context via dual pooling operations (average and maximum), which are subsequently processed by a shared multi-layer perceptron (MLP) (Touvron et al., 2022) under a 16 × dimensionality reduction:


$$\begin{array}{l} {\text{M}}_{c}=\sigma (\text{MLP}\left(\text{AvgPool}\left(\text{F}\right)\right)+\text{MLP}\left(\text{MaxPool}\left(\text{F}\right)\right)), \end{array}$$ (7)

resulting in the channel-weighted feature **F**_*c*_ = **M**_*c*_⊙**F**. Building upon this, the spatial attention sub-module generates a spatial importance map by applying a 7 × 7 convolutional kernel to the concatenated channel-wise pooling descriptors:


$$\begin{array}{l} {\text{M}}_{s}=\sigma ({\text{f}}^{7\times 7}(\left[\text{AvgPoo}{\text{l}}_{c}\left({\text{F}}_{c}\right);\text{MaxPoo}{\text{l}}_{c}\left({\text{F}}_{c}\right)\right])). \end{array}$$ (8)

The final output, **F**_CBAM_ = **M**_*s*_⊙**F**_*c*_, facilitates the adaptive emphasis of critical neuroanatomical structures and discriminative channels. As quantified in the ablation study, the integration of CBAM alone improves the PSNR by 0.41 dB over the baseline, while adding merely 0.012M parameters. This underscores the effectiveness of CBAM in bolstering the structural fidelity of the synthesized 7T images with minimal parameter overhead.

#### 2.2.4 Parameter efficiency

The parameter breakdown of LiteMamba-Synth is summarized in Table 1. The total number of trainable parameters is strictly constrained to approximately 2.15M. The architecture adopts a hierarchical channel scaling strategy from 28 to 224, with the bottleneck contributing the largest share (1.030M). The encoder levels 1 to 3 contribute 0.018M, 0.081M, and 0.317M parameters, respectively, while the decoder levels 3 to 1 contribute 0.361M, 0.092M, and 0.024M. The Wavelet-Enhanced Skip connections (WES) introduce only 0.001M parameters, and the Convolutional Block Attention Modules (CBAMs) are seamlessly integrated within the ConvMamba blocks, adding negligible overhead. Collectively, these plug-and-play enhancements constitute less than 1% of the total parameter budget, successfully validating the exceptional efficiency of the proposed lightweight design.

**Table 1:** Parameter breakdown of LiteMamba-Synth.

Stage	Components	Params (M)
Input stem	Conv2d(1 → 28, *k* = 3)	0.0003
Encoder L1	ConvMambaBlock × 2 (dim = 28)	0.0180
Encoder L2	Downsample (28 → 56)	0.0140
	ConvMambaBlock × 2 (dim = 56)	0.0670
Encoder L3	Downsample (56 → 112)	0.0560
	ConvMambaBlock × 2 (dim = 112)	0.2610
Bottleneck	Downsample (112 → 224)	0.2260
	ConvMambaBlock × 2 (dim = 224)	1.0300
Decoder L3	Upsample (224 → 112)	0.1000
	ConvMambaBlock × 2 (dim = 112)	0.2610
Decoder L2	Upsample (112 → 56)	0.0250
	ConvMambaBlock × 2 (dim = 56)	0.0670
Decoder L1	Upsample (56 → 28)	0.0060
	ConvMambaBlock × 2 (dim = 28)	0.0180
Enhancements	WES modules (3 locations)	0.0010
	(CBAM integrated within ConvMamba)	(Included above)
Output head	Conv2d (28 → 1, *k* = 3)	0.0003
**Total**		**2.1500**

The proposed network contains only 2.15M trainable parameters, demonstrating excellent parameter efficiency for 3T-to-7T MRI translation.

## 3 Experiment

### 3.1 Implementation details

All experiments are conducted on the UNC T1w dataset under a standardized evaluation protocol. The optimization of LiteMamba-Synth is guided by a multi-objective composite loss function that integrates Smooth ℓ_1_ loss, Structural Similarity (SSIM) loss, and an edge-aware Sobel loss. During the initial training phase, these components are weighted as 0.5, 1.0, and 0.5, respectively. To prioritize structural integrity and perceptual fidelity in later stages, the weights are dynamically recalibrated to 0.1, 2.0, and 1.0 beyond the 100th epoch. The training process spans 300 epochs using the AdamW optimizer (Loshchilov and Hutter, 2019). The learning rate is initialized at 1 × 10^−4^ and follows a cosine annealing schedule down to 1 × 10^−6^, preceded by a 5-epoch linear warm-up period. Gradient clipping with a maximum norm of 1.0 is applied to ensure numerical stability. All models are trained on dual NVIDIA RTX 3090 GPUs using Distributed Data Parallel (DDP) with a per-GPU mini-batch size of 2.

### 3.2 Comparison with existing methods

**Figure 3:**
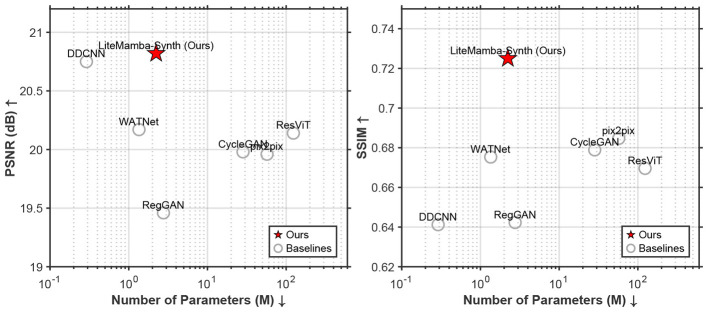
Performance vs. efficiency comparison.

We conducted a quantitative comparison between LiteMamba-Synth and several representative MRI synthesis methods, and the results are summarized in Table 2, Figure 3.

**Table 2:** Quantitative comparison across four synthesis tasks on UNC and BNU datasets.

Method	UNC T1w			UNC T2w			BNU T1w			BNU T2w			Params (M)
	PSNR↑	SSIM↑	RMSE↓	PSNR↑	SSIM↑	RMSE↓	PSNR↑	SSIM↑	RMSE↓	PSNR↑	SSIM↑	RMSE↓	
CycleGAN (Zhu et al., 2017)	19.98	0.679	0.101	24.88	0.754	0.059	21.93	0.788	0.081	26.30	0.805	0.049	28.29
pix2pix (Isola et al., 2017)	19.96	0.685	0.102	24.74	0.753	0.059	22.74	0.810	0.074	26.47	0.818	0.048	57.18
DDCNN (Zhang et al., 2018)	20.75	0.641	0.092	24.21	0.729	0.064	23.02	0.773	0.072	26.88	0.823	0.046	**0.29**
WATNet (Qu et al., 2020)	20.17	0.675	0.099	23.07	0.667	0.072	21.13	0.732	0.089	26.13	0.763	0.050	1.35
RegGAN (Kong et al., 2021)	19.46	0.642	0.107	23.97	0.699	0.065	22.10	0.790	0.079	25.61	0.770	0.053	2.76
ResViT (Dalmaz et al., 2022)	20.14	0.670	0.099	23.47	0.714	0.069	22.78	0.807	0.073	26.58	0.825	0.048	123.44
PTNet (Zhang et al., 2022)	20.82	0.714	0.092	24.66	0.750	0.060	22.98	0.790	0.072	27.34	0.824	0.044	28.13
FS-RWKV (Lei et al., 2025)	**21.01**	**0.726**	**0.090**	**25.31**	**0.781**	**0.057**	**23.36**	**0.839**	**0.069**	27.49	**0.862**	0.043	45.40
**LiteMamba-Synth**	20.82	0.711	0.092	24.85	0.757	0.059	22.99	0.812	0.072	**27.66**	0.824	**0.042**	2.15

**Best** results are in bold, and second best are underlined. LiteMamba-Synth achieves competitive performance with only 2.15M parameters.

On the UNC T1w benchmark, LiteMamba-Synth achieves a PSNR of 20.82 dB, an SSIM of 0.711, and an RMSE of 0.092. Although FS-RWKV attains the highest overall performance, our method matches the PSNR of PTNet while requiring only 2.15M parameters, substantially fewer than PTNet (28.13M) and FS-RWKV (45.40M).

On the UNC T2w task, LiteMamba-Synth obtains a PSNR of 24.85 dB, an SSIM of 0.757, and an RMSE of 0.059, demonstrating competitive performance among recent lightweight and transformer-based approaches. Similar results are observed on the BNU T1w dataset, where LiteMamba-Synth achieves a PSNR of 22.99 dB and an SSIM of 0.812, ranking second in SSIM while maintaining a compact model size.

On the BNU T2w benchmark, LiteMamba-Synth achieves the highest PSNR of 27.66 dB and the lowest RMSE of 0.042 among all compared methods. These results indicate that the proposed architecture can effectively preserve image structures and reduce reconstruction errors while maintaining high parameter efficiency.

Compared with conventional GAN-based methods, LiteMamba-Synth improves the PSNR by 0.84 dB and 0.86 dB over CycleGAN and pix2pix, respectively, on the UNC T1w benchmark. Meanwhile, the proposed model contains only 2.15M parameters, corresponding to reductions of approximately 95.3%, 92.4%, 96.2%, and 98.3% compared with FS-RWKV, PTNet, pix2pix, and ResViT, respectively.

Although DDCNN uses fewer parameters (0.29M), its performance is consistently lower across most evaluation metrics. Overall, the results suggest that LiteMamba-Synth provides a favorable balance between reconstruction quality and model complexity, making it a practical choice for 3T-to-7T MRI synthesis.

#### 3.2.1 Cross-site and cross-modality generalization

To further evaluate the generalization capability of LiteMamba-Synth, we examine its performance across different acquisition sites (UNC and BNU) and imaging modalities (T1w and T2w). As summarized in Table 2, LiteMamba-Synth consistently achieves competitive performance across all four synthesis tasks. Notably, on the BNU T2w dataset, the proposed method achieves the highest PSNR of 27.66 dB and the lowest RMSE of 0.042 among all compared methods. On the remaining benchmarks, LiteMamba-Synth consistently ranks among the leading approaches while maintaining a substantially smaller parameter budget than most competing methods. These results suggest that the proposed lightweight architecture generalizes well across different imaging protocols and acquisition sites without sacrificing reconstruction quality.

##### 3.2.1.1 Qualitative analysis

**Figure 4:**
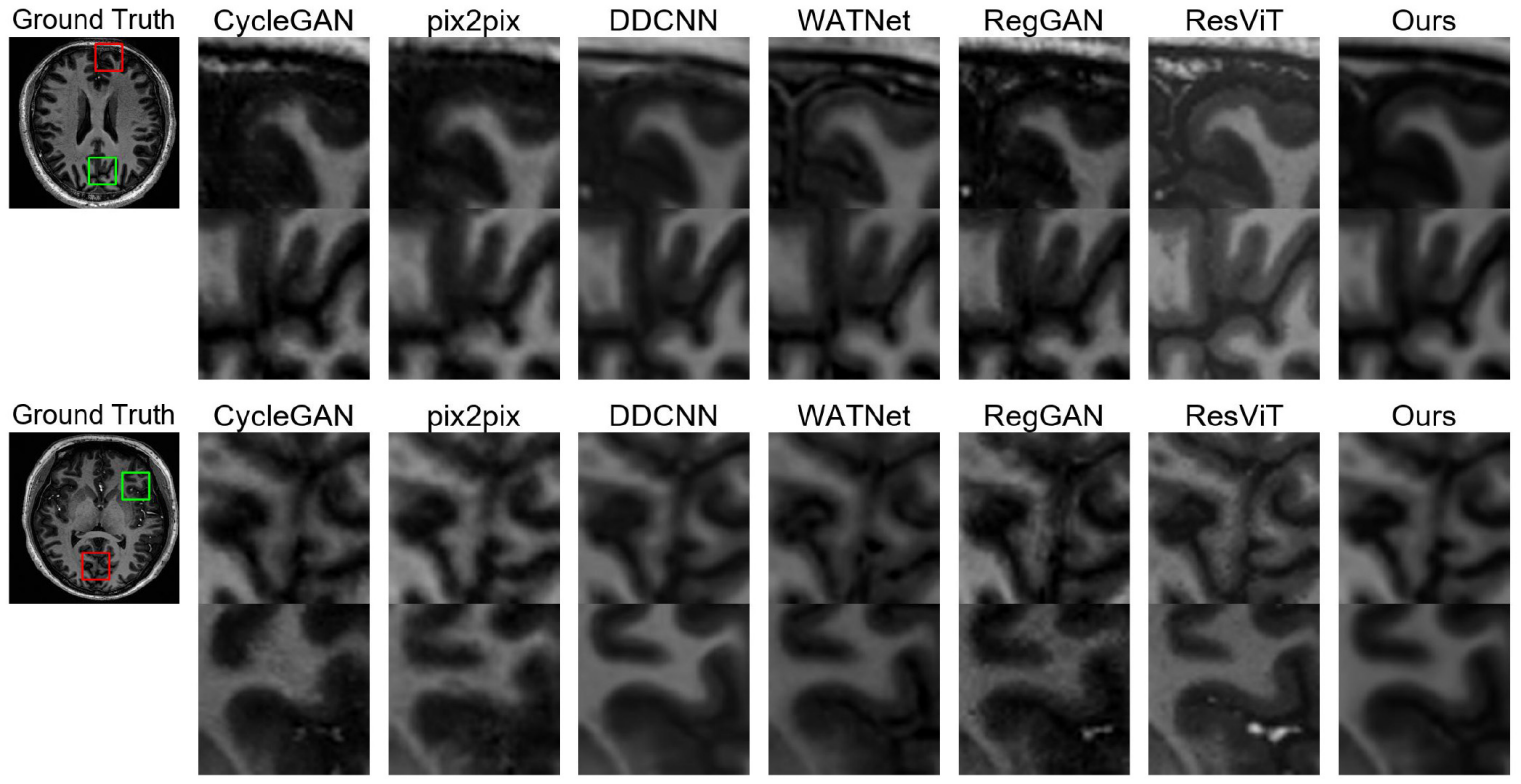
Comparison of image reconstruction performance on images from UNC T1w dataset.

Figure 2 presents representative visual comparisons on the UNC T1w test set. Compared with CycleGAN and pix2pix, which exhibit noticeable over-smoothing around cortical boundaries and white matter regions, LiteMamba-Synth better preserves fine anatomical structures and tissue details. DDCNN retains coarse structural information but produces less realistic textures. In contrast, the proposed method generates reconstructions that more closely resemble the Ground Truth, particularly around the gray-white matter junction highlighted by the red arrows. The enlarged regions further demonstrate the effectiveness of LiteMamba-Synth in recovering subtle anatomical details and preserving structural continuity. Figure 4 illustrates the relationship between reconstruction performance and model size, showing that LiteMamba-Synth achieves a favorable balance between synthesis quality and parameter efficiency.

### 3.3 Ablation analysis

#### 3.3.1 Impact of model capacity

To investigate the effect of model capacity on synthesis performance, we evaluated three configurations with different embedding dimensions, as summarized in Table 3. Increasing the embedding dimension from 24 to 28 improves the PSNR from 20.12 dB to 20.82 dB and the SSIM from 0.696 to 0.711, indicating that a moderate increase in model capacity benefits feature representation and reconstruction quality. However, further increasing the embedding dimension to 32 does not lead to additional improvements. Instead, the PSNR decreases to 20.56 dB and the SSIM drops to 0.695, despite the parameter count increasing from 2.15M to 2.80M. This observation suggests that simply enlarging the model capacity does not necessarily improve generalization under the current training setting. Overall, the configuration with an embedding dimension of 28 achieves the best trade-off between reconstruction performance and model complexity, and is therefore adopted as the default setting of LiteMamba-Synth.

**Table 3:** Ablation study on the impact of model capacity.

Configuration	Embed dim	PSNR (dB)↑	SSIM↑	Params (M)
ConvMamba (baseline)	24	20.120	0.696	1.570
**LiteMamba-Synth**	**28**	**20.820**	**0.711**	**2.150**
LiteMamba-Synth (large)	32	20.560	0.695	2.800

All configurations are evaluated on the UNC T1w dataset using full-resolution 256 × 256 images. Bold values indicate the best performance among the three capacity configurations.

#### 3.3.2 Influence of evaluation region

To investigate the influence of the evaluation region, we further computed quantitative metrics on both the full-resolution 256 × 256 images and the central 128 × 128 cropped regions, as summarized in Table 4. All test images were resized to 256 × 256 during preprocessing. Compared with the full-image evaluation, the central-region evaluation yields higher reconstruction accuracy, with PSNR increasing from 20.820 dB to 23.870 dB, SSIM improving from 0.711 to 0.719, and RMSE decreasing from 0.092 to 0.064. This observation suggests that LiteMamba-Synth reconstructs the anatomically informative brain regions more accurately than peripheral areas. Since the central crop contains a larger proportion of brain tissues and less background content, the resulting metrics better reflect the reconstruction quality of clinically relevant structures. These results indicate that LiteMamba-Synth is particularly effective at preserving anatomical details within the brain region, while maintaining competitive performance over the entire image.

**Table 4:** Impact of evaluation region on quantitative metrics.

Evaluation region	PSNR (dB)↑	SSIM↑	RMSE↓
Full 256 × 256 image	20.820	0.711	0.092
Central 128 × 128 crop	**23.870**	**0.719**	**0.064**

All metrics are computed using the same LiteMamba-Synth model (embedding dimension = 28) on the UNC T1w test set. Cropping the central region yields improved fidelity due to reduced background influence. Bold values indicate the best performance between the two evaluation regions.

#### 3.3.3 Module-wise ablation

To evaluate the contributions of the Wavelet-Enhanced Skip connections (WES) and CBAM modules, we conducted a module-wise ablation study on the UNC T1w dataset, as summarized in Table 5. The baseline model without WES and CBAM achieves a PSNR of 20.17 dB, an SSIM of 0.684, and an RMSE of 0.099. Introducing WES alone leads to a slight performance degradation, yielding a PSNR of 20.15 dB and an SSIM of 0.671, which suggests that indiscriminate high-frequency amplification without attentional guidance may introduce structural noise rather than improving fidelity. In contrast, incorporating CBAM alone improves the PSNR to 20.58 dB and the SSIM to 0.703, demonstrating the effectiveness of attention-based feature refinement. When WES and CBAM are jointly incorporated, LiteMamba-Synth achieves the best overall performance, reaching 20.82 dB PSNR, 0.711 SSIM, and 0.092 RMSE. Compared with the baseline configuration, this corresponds to improvements of 0.65 dB in PSNR and 0.027 in SSIM, while increasing the parameter count by only 0.022M (from 2.128M to 2.150M). It is worth noting that WES alone introduces merely 0.001M additional parameters, which is negligible at the displayed precision, further confirming its nature as a zero-cost enhancement. These results indicate that neither WES nor CBAM alone is sufficient to achieve optimal performance. Instead, the combination of frequency-aware feature propagation and attention-guided feature refinement provides complementary benefits, leading to more accurate MRI synthesis with only a marginal increase in model complexity.

**Table 5:** Module-wise ablation study on the UNC T1w dataset.

Configuration	PSNR (dB)↑	SSIM↑	RMSE↓	Params (M)
Baseline (no WES, no CBAM)	20.170	0.684	0.099	2.128
+ WES only	20.150	0.671	0.099	2.128
+ CBAM only	20.580	0.703	0.095	2.149
**+ WES + CBAM (LiteMamba-Synth)**	**20.820**	**0.711**	**0.092**	**2.150**

All models use embedding dimension 28 and are evaluated on full 256 × 256 images. Bold values indicate the best performance among the four ablation configurations. The WES-only variant shares the same parameter count as the baseline (2.128M) because WES modules introduce fewer than 0.001M parameters, which is negligible at the displayed precision.

## 4 Discussion

### 4.1 Parameter efficiency and practical implications

Benefiting from the linear-complexity Selective State Space Model (Mamba), LiteMamba-Synth achieves competitive MRI synthesis performance with only 2.15M trainable parameters. Compared with conventional GAN-based and Transformer-based methods, the proposed model substantially reduces parameter count while maintaining comparable reconstruction quality. Such a compact design may help reduce the risk of overfitting when training data are limited and can lower computational requirements during deployment. In addition, the region-based evaluation results indicate that LiteMamba-Synth achieves higher reconstruction accuracy within anatomically informative brain regions, suggesting its effectiveness in preserving clinically relevant structures.

### 4.2 Architectural design analysis

The ablation experiments provide further insight into the effectiveness of the proposed design. While WES alone does not improve quantitative performance, CBAM consistently enhances reconstruction quality. The best results are obtained when both modules are jointly incorporated, suggesting that frequency-aware feature propagation and attention-guided feature refinement provide complementary benefits. Furthermore, the model-capacity study demonstrates that an embedding dimension of 28 offers the best trade-off between model complexity and synthesis performance. Increasing the embedding dimension to 32 results in a performance decline, indicating that larger model capacity does not necessarily translate into better generalization under the current training setting.

### 4.3 Limitations and future work

Several limitations remain. First, the current implementation operates on 2D slices and therefore cannot fully exploit volumetric contextual information available in 3D MRI data. Future work will investigate 3D architectures and multi-slice fusion strategies to better capture inter-slice dependencies. Second, although LiteMamba-Synth demonstrates competitive performance across multiple datasets and imaging modalities, its effectiveness on pathological cases has not yet been systematically evaluated. Future studies involving tumor and lesion datasets, as well as broader multi-center validation, will be necessary to further assess its clinical applicability.

## Data Availability

Publicly available datasets were analyzed in this study. This data can be found here: https://springernature.figshare.com/articles/dataset/UNC_Paired_3T-7T_Dataset/23706033.
